# Study on Effect of Extraction Techniques and Seed Coat on Proteomic Distribution and Cheese Production from Soybean Milk

**DOI:** 10.3390/molecules25143237

**Published:** 2020-07-16

**Authors:** Nadia Al-Saedi, Manjree Agarwal, Wujun Ma, Shahidul Islam, Yonglin Ren

**Affiliations:** 1 College of Science, Health, Engineering and Education, Murdoch University, 90 South Street, Perth 6150, Australia; N.Al-saedi@murdoch.edu.au; 2 Department of Food Science and Biotechnology, Faculty of Agriculture, University of Baghdad, Baghdad 10071, Iraq; 3 Australia China Centre for Wheat Improvement (ACCWI), College of Science, Health, Engineering and Education, Murdoch University, 90 South Street, Murdoch, WA 6150, Australia; W.Ma@murdoch.edu.au (W.M.); S.Islam@murdoch.edu.au (S.I.)

**Keywords:** soybean (*Glycine max*), protein isolation, two-dimensional gel electrophoresis, cheesecloth separation, centrifuge separation, cheese production

## Abstract

Soybean-based food products are a major source of protein. In the present study, proteins in soybean milk from seeds of the cultivar Bunya (*Glycine max*) were extracted using the cheesecloth and the centrifuge methods. The milk was produced through mechanical crushing of both whole and split seeds in water. Following separation by either the cheesecloth or centrifuge, proteins were isolated from the soybean milk by using thiourea/urea solubilisation and then separated them using two-dimensional polyacrylamide gel electrophoresis. The isolated proteins were identified by mass spectrometry. A total of 97 spots were identified including 49 that displayed different abundances. Of the two separation techniques, centrifuge separation gave higher protein extraction and more intense protein spots than cheesecloth separation. Eleven of the β-subunits of β-conglycinin, three of the α-subunits of β-conglycinin, and four of the mutant glycinin showed different levels of abundances between separation techniques, which might be related to subsequent cheese quality. Notably, split-seed soybean milk has less allergenic proteins with four α-subunits of β-conglycinin compared to whole-seed milk with eight of those proteins. The sensory evaluation showed that the cheese produced from split-soybean milk received higher consumer preferences compared to that of whole seed, which could be explained by their proteomic differences. The demonstrated reference map for whole and split-seed soybean milk could be further utilized in the research related to soybean cheesemaking.

## 1. Introduction

Soybeans (*Glycine max*) are a significant source of nutrition for humans and animals. They consist of 40% proteins and 20% oil, including several minerals and vitamins [1]. In Australia, soybeans have been grown as a commercial crop since the 1950s. Soybeans are an important part of Australia’s $2.5 billion oilseed industry and the proteins derived from soy are used in food products such as meals, drinks, and sports beverages. The Australian market offers three types of soy beverages: Asian soybean drinks made with water, beverages made using whole soybean extract mixed with sugar, and drinks made from isolated soy protein added to vegetable oils, minerals, vitamins, and flavors [2]. Soybeans are, therefore, an integral part of Australian food markets.

Soybeans are versatile and can be used for their health benefits and material-enhancement properties. For example, the addition of soy proteins to food decreases cholesterol levels, and, thereby, lowers the risk of cardiovascular disease [3]. Soybean flakes are an excellent aggregate agent for spinning textile fibres after isolating the oils [4].

There are four main types of soybean proteins: 2S, 7S, 11S, and 15S. Seeds of the soybean mostly contain storage proteins such as β-Conglycinin, along with glycinin, which makes up 70–80% of the total protein content. β-Conglycinin is composed of three subunits: the α-subunit, α′-subunit, and β-subunit [5,6]. The remaining 20–30% of proteins include cytochrome c, β-amylase, lipoxygenase, lectin, trypsin, urease, together with inhibitor of Kunitz trypsin (KTI), and an inhibitor of Bowman-Birk (BBI) of chymotrypsin [7].

The two-dimensional polyacrylamide gel electrophoresis (2D-PAGE) technique was used to separate diverse globulin proteins, anti-nutritional proteins, and allergens from soybean seeds [8]. Liquid chromatography-mass spectrometry (LC-MS) is also a powerful method for identifying and characterising protein profiles that could be applied to the soybean. Recent studies that aimed to identify soybean proteins focused on the proteomic analysis of soybean seed proteins and comparing different protein solubilisation methods [9]. For example, research by Natarajan [10] successfully isolated and identified proteins from the soybean embryonic axis. An earlier study successfully extracted and characterised low abundance proteins from soybean seed powder by using different concentrations of isopropanol and analysis by 1D-PAGE and 2D-PAGE [11]. The proteins from soybean samples were extracted via different solubilisation methods, isoelectric precipitation enzymatic extracts, ultrafiltration, electrodialysis, precipitation, and supercritical carbon dioxide extraction and alkaline gradient extraction [12,13,14]. The protein structure was changed by different factors such as extraction techniques, temperature, pH, and ion strength as well as reactions with other components like different proteins, saccharides, or lipids [15,16]. A study by Hojilla [16] found that ultrafiltration followed by diafiltration (UF-DF) of soybean proteins extracted significantly higher values on the solubility and surface hydrophobicity index than did acid-precipitation. Heat treatment affects the extractability of soybean proteins. Crude proteins, which range from 4.52% to 4.84% and come from five different cultivars, were extracted by grinding soybean in the Soymimax machine [17].

Soybean milk was concentrated via a combination of microfiltration and ultrafiltration to produce a soft cheese-like product [18]. In another study, soybean milk was extracted from split-seed with boiled water for 15 min and then filtered through eight layers of cheesecloth [19]. For our present study, since the soybean milk will be used to make cheese, the extraction of proteins by a chemical-free water extraction process is required. Keeping this in mind, both whole and split soybean cultivar Bunya seeds were used. So far, no protein reference map has been reported for split-seed or whole-seed soybean milk. Therefore, the present study compared two different separation techniques - cheesecloth and centrifuge - for extraction of proteins from both split and whole-seed milk. The analysis of proteins in the soybean seed milk was carried out using 2D-PAGE gels, which is followed by LC-MS/MS for protein identification. This study determines the influence of the separation technique on whole-seed and-seed split soybean milk protein content on the process of cheesemaking.

## 2. Results and Discussion

### 2.1. Comprehensive Protein Profile of Soybean Milk

**Figure 1:**
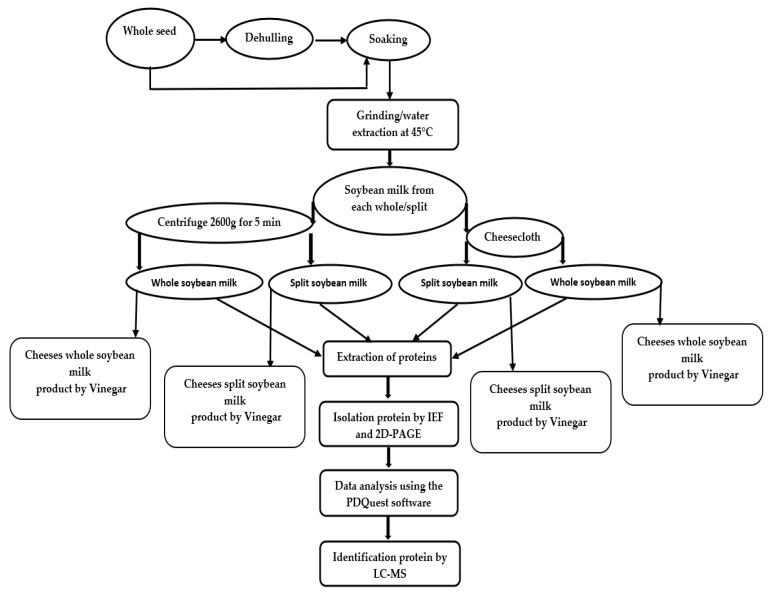
Diagram of workflow for extraction of soybean milk, its analysis, and cheese production.

In the present study, protein extraction from whole soybean seed with seed coats (hulls) and split soybean seeds without seed coat were compared using the cheesecloth and centrifuge methods (Figure 1). The total protein concentration and numbers of protein spots identified by a 2D-PAGE in soybean milk under each condition are shown in Table 1. Both the total protein concentration and total protein spots in centrifugal separation were higher than in cheesecloth separation. Similarly, the whole-seed milk demonstrated higher protein content and total number of protein spots than split-seed milk.

**Figure 2:**
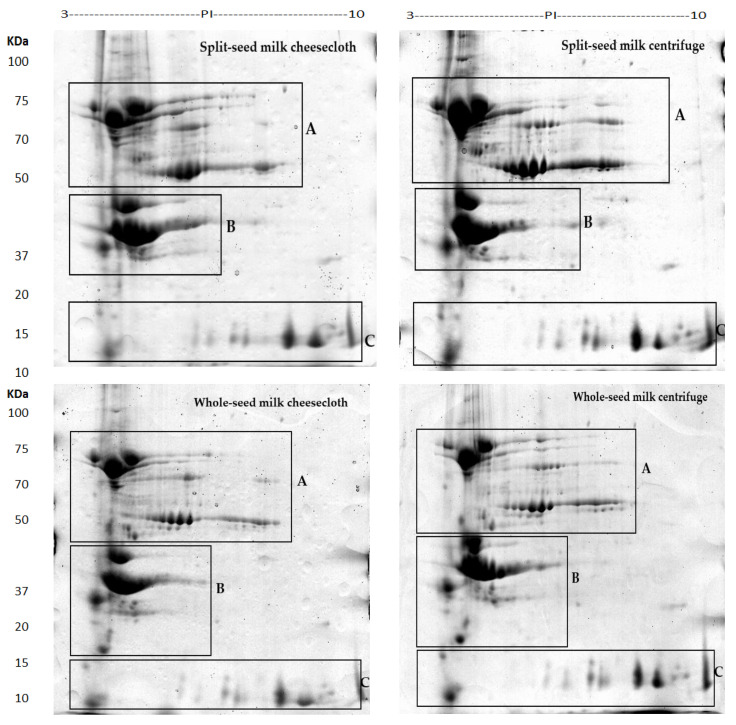
Proteomic comparison of the storage proteins of soybean milk protein from whole and split soybean seeds with a different processing profile of the cultivar Bunya of soybean seeds, as shown by 2-D gel electrophoresis.

A high-resolution image of the extractability of the soybean milk proteins pattern is presented in Figure 2. The results showed that the 2D-PAGE was an efficient approach to investigate the differential abundance of soybean milk. Using PDQuest analysis software with a standard spot number (SSP), the quantity of each spot and standard deviation was calculated, as seen in Table 2. A total of 97 unique protein spots were revealed in the 12 gels, and 49 protein spots had different abundance levels or different protein quantities between the samples.

**Figure 3:**
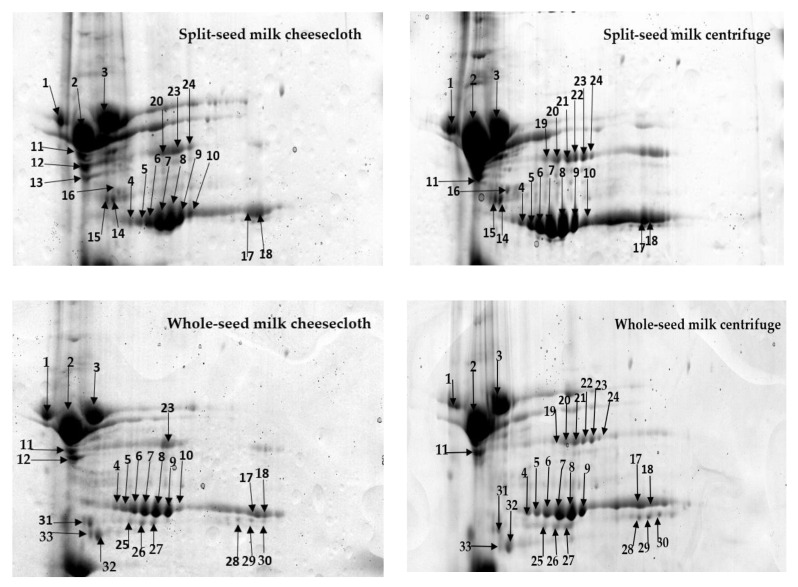
Comparison of a particular zone on the two-dimensional gel describing the abundance of differentiating proteins extractability in soybean milk as influenced by separation techniques and seed coat of the cultivar Bunya of soybean seed. Reference region A presented in Figure 2 are studied.

**Figure 4:**
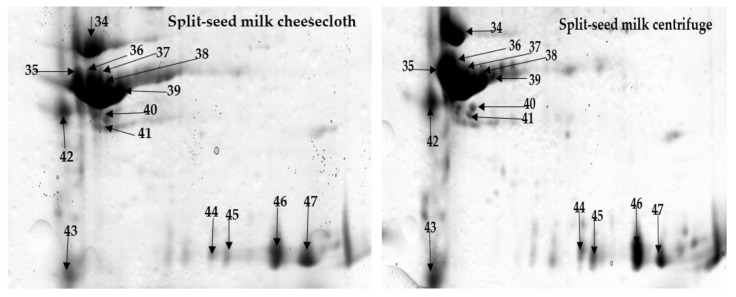

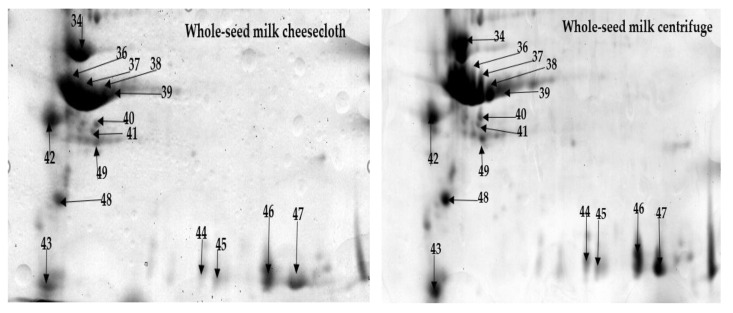
Comparison of a particular zone on the two-dimensional gel describing the abundance of differentiating proteins extractability in soybean milk, as influenced by the seed coat and separation methods of the Bunya cultivar of soybean seeds. Reference region B and C presented in Figure 2 are studied.

Protein spots appeared in three specific positions of the gels (Figure 2, Figure 3 and Figure 4). Some protein spots had similar molecular weights but different isoelectric point (PI) values. These spots might be isoforms obtained from different genes of a multigene family [10]. Several protein spots identified in the whole and split-seed soybean milk separated by centrifuge were notably absent in the milk processed by cheesecloth and vice versa (Table 3 and Table 4). 

The separation techniques clearly impacted protein extractability, and this could further influence the total protein concentration and processing of soybean cheeses (Table 5 and Table 6). The NCBI database accession number of the best match, molecular weight, isoelectric point, percentage sequence coverage, MOWSE score, and matched peptides are displayed in Table 7. In this investigation, 49 proteins were successfully identified in the split-seed and whole-seed soybean milk of which 26 proteins belonged to β-conglycinin and 12 proteins belonged to glycinin proteins. The two main storage proteins in soybean seed, 7S globulins as β-Conglycinin subunits, and 11S globulins are identified through glycinin proteins. Both proteins have different fundamental properties leading to different functional properties [20]. Glycinin was reported to precipitate faster and produce harder tofu gels than β-Conglycinin [20]. Glycinin is composed of five subunits—G1, G2, G3, G4, and G5—among which G1 and all G2 subunits of glycinin are allergen subunits [21]. Each subunit contains acidic (A) and basic (B) chains linked together by disulfide bonds [22]. The G5 subunit showed one acidic polypeptide (spot number 34, Figure 4, and Table 7). The G4 subunit showed two basic polypeptides (spot numbers 46 and 47, Figure 4, and Table 7). The absence of G1 and G2 subunits in cultivar Bunya observed in our study could be due to the absence of the gene(s) encoding. Additionally, split-seed soybean milk has less allergenic proteins compared to whole-seed, which has eight of the α-subunits of β-conglycinin. Hence, our research provided safety for consumers by eliminating the majority of allergenic proteins in soybeans.

Soybean seeds were also found to contain 1% of a sucrose-binding protein. This protein is responsible for binding sucrose to improve cotyledons and is similar to the vicilin-like protein in lupin seeds [23,24].

The various protein spots in gels from cheesecloth and centrifuge-separated milk could correspond to proteins modified during the extraction and separation process. The compound genome of soybeans is expected to comprise multiple copies of many genes and different sequences of amino acids in several isoforms. In the two separation techniques, the differences between acidic and basic polypeptide protein spots in the split and whole soybean milk were mainly found in three regions (Figure 2, Figure 3 and Figure 4), particularly in the pH range of 4–7.

### 2.2. Influence of Separation Techniques and Seed Coat on Protein Extractability Form Soybean Milk

#### 2.2.1. Separation Techniques

The separation techniques have a significant impact on the extractability of soybean milk proteins. For instance, one of the mutant glycine A3B4 (spot number 12, Table 2 and Table 3, Figure 3) was present in both the whole and split-seed milk only when separated with the cheesecloth method. In contrast, two of the sucrose binding protein homolog S-64 (spots numbers 21 and 22) and one of the Glyso Sucrose-binding protein (spot number 19, Table 2 and Table 3, Figure 3) were detected in both whole and split-seed milk only when separated with the centrifuge method. Research by Natarajan [9] found such protein spots in soybean seeds to have a different abundance across four different protein extraction/solubilisation methods with urea, thiourea/urea, phenol, and trichloroacetic acid (TCA)/acetone. However, he did not report α-Subunit of β-Conglycinin (spot number 1).

In addition, three of the α-subunits of β-conglycinin (spot numbers 1, 2, and 3, Table 2 and Table 3, Figure 3) and six of the β-subunits of β-conglycinin (spots numbers 7, 9, 10, 17, 18, and 42, Table 2, Table 3, and Table 7, Figure 3) had a higher level of abundance in both whole and split-seed milk with centrifuge separation than with cheesecloth separation. Similarly, two Glycinin G4 subunits (spots numbers 46 and 47, Table 2 and Table 3, Figure 4) and one uncharacterised protein (spot 43, Table 2 and Table 3, Figure 4) were detected in higher quantities in both whole and split-seed milk with centrifugal separation than with cheesecloth separation. These results could be interpreted to mean that centrifugal separation removed most of the non-proteinaceous components from the supernatant (milk), which resulted in higher extractability of proteins from the soybean milk compared to that of cheesecloth. However, some of the high molecular weight proteins could have a lower density in the milk produced by the centrifugal method compared to cheesecloth. Hence, centrifugal separation provides better extractability. The efficiency of separation of soybean proteins depended on its mass, shape, and density and the speed at which a molecule moves in a centrifugal field [25].

#### 2.2.2. Seed Type (Split vs. Whole)

The presence of the seed coat was found to influence the extractability of proteins in soybean milk. For example, three of the β-subunits of β-conglycinin subunit (spots numbers 14, 15, and 16, Table 2 and Table 4, Figure 3) and one of the uncharacterised proteins (spot number 35, Table 2 and Table 4, Figure 4) were present only in split-seed milk for both separation techniques. These observations are in line with the conclusion of Mooney and Thelen [26] that proteins of soybean seeds were detected as β-subunits of β-conglycinin subunits when robotic automation was used in every step after 2-D gel electrophoresis and identification by peptide mass fingerprinting. On the other hand, four of the α-subunits of β-conglycinin (spots numbers 26, 30–32) and four of the β-subunits of β-conglycinin subunit (spots numbers 25, 27–29) were present only in whole-seed milk from both separation techniques (Table 2 and Table 4, Figure 3). In addition, one of the Glycinin A3B4 subunits (spot number 33, Table 2 and Table 4, Figure 3) and one of the uncharacterised proteins (spot number 48, Table 2 and Table 4, Figure 4) were identified only in whole seeds for both methods. These spots might be different isoforms derived from different genes from the seed coat. The extractability level in both separation methods could also be affected by the presence of seed coats. They are lower in mass and do not catch proteins with the supernatant. As a result, these proteins are highly abundant in whole-seed milk. These results are unlike the previous study [27] that found the lupin seed coat can affect the separation of proteins with a centrifugal method.

Seven of the β-subunits of β-conglycinin subunit (spots numbers 4–10, Figure 3) appeared as a chain in the gels at the same molecular weight, but with different PI values at a significantly higher level of abundance in split-seed soybean milk than in whole-seed soybean milk. These could be involved in phosphorylating post-translationally of a set of proteins in soybean milk [28]. This result was similar to that of Natarajan’s [13] study, which used three types of strips—wide pH 3–10, narrow 4–7, and 6–11—to separate proteins from soybean seeds

### 2.3. Evaluation of Cheese Production

The total protein concentration of soybean cheese from each method is presented in Table 5. The total protein contents of both split-seed and whole-seed cheeses in centrifuge separation were significantly higher than the total protein content of split and whole-seed cheese in cheesecloth separation. Cheese produced from whole-seed milk by centrifuge had slightly better color and flavors compared to that of cheesecloth, which is likely because there is more efficient separation of the non-pretentious object of the seed coats in the centrifugal method.

**Figure 5:**
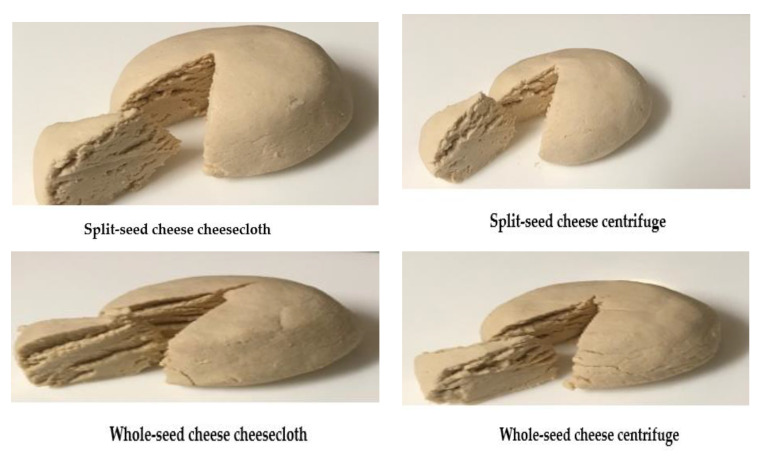
Characteristics of cheeses production from soybean milk.

On the other hand, yields from soybean curds were influenced by separation techniques, as shown in Table 6. For instance, split-seed milk separated by cheesecloth yielded significantly higher curd (*p* < 0.05) than by centrifuge. Furthermore, the yield from cheesecloth separation was very close to the yield from cow’s milk. Similarly, for whole-seed milk, the yield from cheesecloth separation was slightly higher than from centrifugal separation. Panelists appeared to appreciate split-seed cheese from both separation techniques more than whole-seed cheeses. This might be due to the seed coat and the external appearance of the cheese (Table 6 and Figure 5). The protein content was 21.26% in split-seed cheese under cheesecloth filtration, which is similar to the value 21.00% reported in earlier studies using acetic acid in the coagulation of split-seed milk. This was boiled and then filtrated through eight layers of cheesecloth [19].

Good quality split-seed cheese is characterized by a brighter color and smooth texture. Four of the α-Subunit of β-Conglycinin (spots numbers 26 and 30–32) were found only in whole-seed milk, which may change the taste of the whole-seed cheese. The taste, color, and texture of whole-seed cheese may be affected by other components in the seed coat such as dietary fiber. Dust [29] reported that seed coat contains 83.3% total dietary fiber with a ratio of insoluble to soluble fiber of 5.0%. Four mutant glycinin subunits (spots numbers 36–39, Table 2) demonstrated higher levels of abundance in split-seed milk with cheesecloth separation. In contrast, eight of β-Conglycinin subunits (spots numbers 25–32, Table 2 and Table 7) were absent in split-seed milk. Therefore, these results led to the suggestion that split-seed cheese texture is possibly improved by a high abundance of glycinin subunits or a high glycinin /β-Conglycinin subunits ratio. The 11S glycinin proteins/ 7S β-Conglycinin subunits ratio in soymilk strongly affected the textural properties of tofu [30]. Glycinin precipitates faster and produces harder tofu gels than β-Conglycinin [20]. A study by Natarajan [31], which used 2D-PAGE with three different immobilised pH gradient (IPG) strips, found that most of the β-Subunits of β-conglycinin were completely separated in the pH range of 3.0–10.0.

However, the same study did not find four of the mutant glycinin subunits (spots numbers 36–39) when using a pH gradient from 4 to 7.0 in the first dimension. β-Conglycinin proteins were identified as a genotype in cultivars of soybeans including β-Conglycinins with two mRNA groups [31]. The first mRNA group encodes α and α’β-conglycinin subunits. Additionally, the second mRNA group encodes the β-subunit of β-conglycinin [32]. The main proteins in soybean seeds are conglycinin, which are comprised of an α subunit, α’ subunit of β-conglycinin, and β-subunits of β-conglycinin [33]. Only the α subunit of β-conglycinin is detected to be allergenic [34]. However, the major storage proteins in the soybean milk were identified as β-subunits of β-conglycinin and Glycinin proteins with different levels of abundances between separation techniques. These results further indicate that the protein components play an essential role in the formation of soybean cheese.

## 3. Materials and Methods

### 3.1. Chemicals

Chemicals for electrophoresis including sodium dodecyl sulfate (SDS), *N*,*N*,*N*_,*N*_-tetramethylethylendiamine (TEMED), ammonium persulfate, thiourea, urea, dithiothreitol (DTT), CHAPS, glycerol, and Tris–HCl (pH 8.8) were purchased from Sigma (Willetton, WA, Australia). IPG strips with (pH 3–10), 17-cm catalogue # 163-2009, and 40% acrylamide/bis solution ampholytes (pH 3–10) were purchased from Bio-Rad (Gladesville, New South Wales, Australia). All chemicals were standard reagent grade laboratory chemicals. Water from a Sartorius reverse osmosis system (Göttingen, Germany) was used for all solutions.

### 3.2. Plant Materials and Preparation of Soybean Milk

Soybean seeds of the cultivar Bunya (*Glycine max*) were sourced from PB Agrifood (Wilsonton, Queensland). The tested soybean samples were newly harvested (2019) pesticide-free seeds stored at −20 °C until use.

For preparation of split seeds, the seeds were broken into halves and seed coats were removed with mortar and pestle. Ten grams of each dry half split and whole seed were soaked separately in water overnight with a ratio of 1:3 soybean: water at room temperature (24 ± 1 °C). A stainless-steel gas-tight blender (250 mL), fitted with a screw-top lid containing a septum, was used for the grinding of soaked samples. Ten grams of each wet split and whole seed were ground separately with 100 mL of water maintained at a temperature of 45 °C. The mixes were divided into two equal parts each. One half was separated using four layers of cheesecloth, and the other half was separated using a centrifuge from Qingdao Xinya Aipu Electric Appliance (AIPU) at 2600× *g* for 5 min. The filtrates were stirred to get the final volume of soybean milk. The preparation of the milk was done in triplicate using the same procedure with three different lots of seeds. The workflow diagram is shown in Figure 1.

### 3.3. Extraction of Protein

Four types of soybean milk from cheesecloth and centrifuge were used for extracting the proteins. The protein was precipitated by mixing 400 µl of the soybean milk with 1600 µL of ice-cold acetone at −20 °C overnight. The precipitate was collected by centrifugation at 13,000× *g* for 10 min. The protein pellet was dissolved in rehydration buffer (7M urea, 2M thiourea, 4% 3-[(3-cholamidopropyl) dimethylammonio]-1-propanesulfonate (CHAPS), 65 mM DTT, and 2% IPG buffer (mixing two kinds of acrylamide mixture, one with Immobiline having acidic buffering property and other with basic buffering property). The samples were incubated for 4–5 h at room temperature. Lastly, the protein concentration was determined by using reducing agent and detergent compatible (RC DC) protein assay kit (Bio-Rad, Herculles, CA) and a Lambda 25 UV–vis spectrometer (PerkinElmer). Based on the calibration curve, 900 µg of lupin milk protein was loaded onto IPG strips for each sample.

### 3.4. Two-Dimensional Gel Electrophoresis and Data Analysis

The protein was separated by iso-electric focusing (IEF) on 17 cm IPG strips with pH 3–10, which were rehydrated with the buffer (7 M urea, 2 M thiourea, 2% CHAPS, 65 mM DTT, and 2% IPG buffer) containing 900 µg of protein. The strips were focused at 250 V for 1 h, 1000 V for 1 h, 10,000 V for 5 h, 70,000 V for 1 h, and 500V for 48 h, at 20 °C using Protein Isoelectric focusing (IEF) cell (Bio-Rad). The gel strips were incubated with equilibration buffer (50 mM Tris–HCl [pH 8.8], 6 M urea, 30% (*v*/*v*) glycerol, 2% (*w*/*v*) SDS, and 0.002% bromophenol blue, containing 65 mM DTT) for 15 min and another 10 min by replacing DTT with 135 mM iodoacetamide in the same buffer and, subsequently, placed onto 12% acrylamide/bis (31.5:1) gels, using Protean II Xi cell (Bio-Rad). Strips were overlaid with agarose sealing solution (1% agarose and 0.002% bromophenol) and running buffer consisting of 2.5 mM Tris–Base, 19.2 mM glycine, and 0.01% SDS. The 2D-PAGE gels were visualised using the Coomassie Brilliant Blue (CBB) staining method. Three biological replications were run three times with individual extractions and IEF.

The gels were imaged and analyzed using PDQuest (Bio-Rad) to investigate quantitative changes in cellular protein abundance. The protein spots from triplicate gels of each separation methods were matched to each other and compared to an image called a ‘master gel or ‘match set standard.’ The master gel includes all the information about the spots in all gels matched. The spots that were quantitatively and statistically significant were compared using analysis sets. The quantification of individual spots was recognised with a unique standard spot number (SSP) that provides the location of the spot. Statistical analysis of the data was carried out using Microsoft Excel 365, 2019. The compared means of quantity and standard deviation (Sd) were calculated from three spots in different gels by International Business Machines Corporation, Statistical Product and Service Solutions (IBM SPSS) statistics 24 version.

### 3.5. Identification of Protein

The protein spots were manually picked from Coomassie Brilliant Blue stained two-dimensional gels and further analysed by mass spectrometric peptide sequencing. The spots were analysed by Proteomics International Ltd. Pty, UWA, Perth, Australia. Protein samples were digested with trypsin and peptides were extracted with standard techniques [35]. Peptides were analysed by LC-MS using the Agilent 1260 infinity HPLC system coupled to an Agilent 1260 Chipcube Nanospray interface on an Agilent 6540 mass spectrometer. Tryptic peptides were loaded onto a ProtID-Chip-150 C18 column (Agilent) and separated with a linear gradient of water/acetonitrile/0.1% formic acid (*v*/*v*). The software Mascot (Matrix Science) with a taxonomy set to Viridiplantae (Green Plants) was used to identify proteins. The search parameters for LC-MS/MS on the Agilent 6540 mass spectrometer were with peptide tolerance of ±0.2. The peptide charges were set at 2+, 3+, and 4+ and 1 missed cleavage with a significance threshold at *p* < 0.05. Generally, a match was accepted where two or more peptides from the same protein were present in a protein entry in the Viridiplantae database. The peptides have already been matched to proteins at a higher level-of-significance analysis against an alternative database or further de novo peptide sequencing. Protein identification was completed by searching the National Centre for Biotechnology Information (NCBI) nonredundant database using the Mascot search engine.

### 3.6. Determination of Acetic Acid Volume in Vinegar

The acidity of the vinegar was determined by titrating 5 mL of vinegar and 25 mL of distilled water with 0.1N NaOH solution and using phenolphthalein as an indicator with a pink color as an endpoint [36]. Change in pH was measured using an Orion Dual Star pH meter.

### 3.7. Fermentation of Milk to Obtain Soybean Cheeses

A total of 10 L of the whole and split-seed milk were taken. Cow’s milk was used as a control. Each 10 L was divided into equal parts. One portion of split-seed or whole-seed milk was separated using cheesecloth, and the other fraction was separated by centrifuge. Each mixture was heated to 80 °C. The milk was then divided into two equal parts with each part further duplicated to 2.5 L for each batch. Subsequently, 2% (*v*/*v*) of vinegar with titratable acidity of 7.80% (expressed as acetic acid) was added slowly until a pH of 5, which is the isoelectric point for soybean milk/cow’s milk, was reached. At this stage, white clouds on a yellow serum could be visualised. Each mixture of curd and whey was poured through a sieve covered with cheesecloth for the drainage of whey. The curd was weighed and salt (2%) was added. Then it was pressed for 10 h at 4 °C and packed. The workflow diagram is shown in Figure 1.

### 3.8. Determination of Curd Yield of Cheese

The yield of cheese was determined by using the following equation.

$$\mathrm{Yield}\;\mathrm{of}\;\mathrm{cheese}\;(w/v)\;\%\;=\frac{X2}{X1}$$

where:

X_1_ = Volume (mL) of soybean milk
X_2_ = Weight (g) of protein coagulant (soybean curd)

### 3.9. Determination of Total Protein in Soybean Milk and Cheese

AOAC (2000) methods were used to estimate the protein (N × 5.7) contents (method 981.10C) [37].

### 3.10. Sensory Evaluation 

The samples were examined at room temperature 22 ± 2 °C by 30 panelists including staff and students of the department. The samples were arranged in a randomised order in plastic containers. The panel was asked to evaluate four types of soybean cheese and cow’s milk cheese with 1-week storage at 4 °C for appearance, color, flavor, and texture, using a 20-point hedonic scale (5-excellent, 4-good, 3-satisfactory, 2-less satisfactory, 1-unsatisfactory) [38]. Outcomes were statistically analysed using SPSS Version 24 software. One-Way Analysis of Variance (ANOVA) was used to determine the statistical differences between the sample means with the level of significance set at *p* < 0.05 or 0.01.

## 4. Conclusions

This is the first study comparing the effect of separation methods on the protein profiles of the whole-seed and split-seed soybean milk using the proteomic tools 2D-PAGE and MS. At the milk production stage, the centrifuge method appeared as a better option to provide higher protein concentration than cheesecloth. However, cheese production was heavily influenced by the seed coat that masked the influence of a separation technique, which was particularly true in the case of split seeds. Cheese produced from the split-seed milk with a cheesecloth separation method achieved the preference of the sensory panelists and relatively higher yield, which is speculated to be attributed to the higher abundance of glycinin content or a high glycinin/β-Conglycinin subunits ratio. In addition, this study showed a reduction of allergenic proteins in split-seed soybean milk compared to that of whole-seed since, out of eight of the α-subunits of β-conglycinin detected in whole-seed milk, only four appeared in split-seed milk. This finding indicated that, in the cheese production process, more emphasis was given on the protein components rather than only protein content.

## Figures and Tables

**Table 1:** List of the total protein concentration and numbers of protein spots detected by PDQuest software from two-dimensional polyacrylamide gel electrophoresis (2D-PAGE) of soybean milk of each condition.

Type of Soybean Seeds to Make Milk	Separation Method	Total Protein (g/100 mL) Mean ± SD (n = 3)	Spots Numbers Mean ± SD (n = 3)
Split	cheesecloth	2.03 ± 0.15	73 ± 1.70
Split	centrifuge	2.56 ± 1.00	80 ± 1.50
Whole	cheesecloth	2.60 ± 0.10	81 ± 1.52
Whole	centrifuge	2.97 ± 0.05	93 ± 0.50

SD = standard deviation. Number of replicates (n = 3).

**Table 2:** Quantitative list of differentially abundant protein spots with respect to extractability in the soybean milk made from different sample types and separation methods. The spots are significantly different (*p* < 0.05) at PDQuest Bio-Rad.

		Split-Soybean Milk		Whole-Soybean Milk	
		Cheesecloth	Centrifuge	Cheesecloth	Centrifuge
Spot No	SSP	Mean ± SD (n = 3)	Mean ± SD (n = 3)	Mean ± SD (n = 3)	Mean ± SD (n = 3)
1	2701	207.91 ± 1.00	279.66 ± 0.20	136.77 ± 0.93	236.62 ± 0.61
2	2706	1275.11 ± 1.00	1539.53 ± 2.05	70.51 ± 0.61	403.36 ± 0.75
3	3704	226.53 ± 1.03	620.87 ± 0.56	207.06 ± 0.52	446.47 ± 1.42
4	4501	72.46 ± 0.18	150.61 ± 0.54	69.47 ± 0.56	60.13 ± 0.14
5	4502	136.92 ± 0.61	188.12 ± 0.99	115.00 ± 0.58	90.60 ± 0.54
6	4503	185.06 ± 0.25	334.73 ± 0.17	122.41 ± 0.38	58.98 ± 0.05
7	4507	181.52 ± 0.51	648.99 ± 4.93	59.93 ± 0.14	178.55 ± 0.50
8	5509	573.21 ± 0.19	694.61 ± 0.60	253.94 ± 0.57	57.89 ± 0.10
9	5510	111.06 ± 0.56	257.23 ± 0.21	50.55 ± 0.49	81.47 ± 0.43
10	5507	185.45 ± 0.40	266.56 ± 0.47	20.03 ± 0.61	34.26 ± 1.74
11	2707	20.40 ± 0.01	177.26 ± 0.54	25.81 ± 0.56	132.56 ± 1.00
12	2704	58.40 ± 0.01	ND	137.48 ± 0.59	ND
13	2601	43.20 ± 1.16	ND	ND	ND
14	3601	81.29 ± 0.58	38.97 ± 0.57	ND	ND
15	3603	73.72 ± 0.02	69.60 ± 0.43	ND	ND
16	3602	31.74 ± 1.74	24.20 ± 0.56	ND	ND
17	6503	115.94 ± 0.97	144.84 ± 0.56	44.06 ± 1.02	126.23 ± 1.08
18	6504	245.25 ± 0.99	304.51 ± 1.15	97.22 ± 0.59	152.83 ± 0.57
19	5703	ND	85.52 ± 0.05	ND	21.72 ± 0.62
20	5704	60.31 ± 0.01	98.30 ± 0.05	ND	112.29 ± 0.05
21	5701	ND	284.05 ± 0.60	ND	58.51 ± 0.05
22	5702	ND	171.01 ± 0.58	ND	80.54 ± 0.01
23	5705	85.42 ± 0.01	263.02 ± 0.58	38.40 ± 0.05	84.06 ± 0.57
24	5706	49.85 ± 0.44	137.67 ± 0.01	ND	28.25 ± 0.57
25	4508	ND	ND	20.40 ± 0.01	30.06 ± 1.02
26	4504	ND	ND	38.48 ± 0.59	77.38 ± 0.60
27	4501	ND	ND	26.54 ± 0.58	41.96 ± 0.91
28	6506	ND	ND	18.80 ± 0.21	49.43 ± 0.58
29	6507	ND	ND	21.43 ± 0.57	47.36 ± 0.01
30	6508	ND	ND	20.50 ± 0.63	39.69 ± 0.27
31	3501	ND	ND	202.99 ± 0.56	102.19 ± 0.57
32	3404	ND	ND	89.65 ± 0.05	77.24 ± 0.22
33	3502	ND	ND	75.21 ± 0.57	47.53 ± 0.57
34	3402	163.15 ± 0.15	41.74 ± 0.56	287.28 ± 1.15	44.96 ± 0.59
35	2302	526.88 ± 0.58	17.55 ± 0.28	ND	ND
36	2308	661.08 ± 0.87	75.98 ± 0.01	200.21 ± 0.37	184.68 ± 0.68
37	2309	393.39 ± 0.35	42.43 ± 0.49	320.79 ± 0.29	97.55 ± 0.07
38	3309	1408.00 ± 1.15	151.47 ± 0.56	657.14 ± 0.14	183.00 ± 1.12
39	3308	997.10 ± 0.57	231.30 ± 1.61	828.98 ± 0.57	668.05 ± 0.04
40	3306	66.04 ± 0.60	109.10 ± 0.58	93.06 ± 0.02	150.44 ± 0.58
41	3305	25.56 ± 0.05	132.41 ± 0.58	44.71 ± 0.35	140.45 ± 0.04
42	2301	551.87 ± 0.58	667.58 ± 0.72	691.18 ± 0.59	959.22 ± 0.02
43	2207	23.44 ± 0.01	82.64 ± 0.58	45.44 ± 0.05	86.03 ± 0.58
44	6302	68.52 ± 0.02	195.52 ± 0.05	40.93 ± 0.58	156.74 ± 0.58
45	6301	31.26 ± 0.56	169.77 ± 0.37	20.87 ± 0.67	31.10 ± 0.55
46	7301	731.75 ± 0.57	838.44 ± 0.57	497.53 ± 0.55	1123.50 ± 0.62
47	7208	486.16 ± 0.57	690.42 ± 0.01	24.18 ± 0.34	751.49 ± 0.57
48	2304	ND	ND	235.98 ± 0.58	364.00 ± 0.61
49	3302	ND	ND	59.61 ± 0.01	88.25 ± 0.49

SSP = standard spot number. SD = standard deviation. Number of replicates (n = 3). ND = not detected.

**Table 3:** Proteins extractability in soybean milk as affected by separation.

Type of Seeds to Make Milk	Separation Techniques	Present Only in Cheesecloth Separation	Higher Level of Abundance in Cheesecloth Separation *	Present Only in a Centrifuge	Higher Level of Abundance in Centrifuge Separation *
Split	Cheesecloth versus centrifuge	12 [Mutant glycinin A3B4] 13 [Uncharacterized protein]	14–16, 42 [β-Subunit of β-Conglycinin] 34 [Glyg5_SoybnGlycinin] 35 [Uncharacterized protein] 36–39 [Mutant glycinin Subunit A1aB1b]	19 [Glyso Sucrose-binding protein] 21, 22 [Sucrose binding protein homolog S-64]	1–3 [α-Subunit of β-Conglycinin] 4–11, 17, 18, 24, 42 [β-Subunit of β-Conglycinin] 20, 23 [Glyso Sucrose-binding protein] 40, 41 [Glyso Lectin] 45 [Glyso Glycinin] 46, 47 [Glycinin G4 subunit] 44 [Mutant glycinin A3B4] 43 [Uncharacterized protein]
Whole	cheesecloth versus centrifuge	12 [Mutant glycinin A3B4]	4–6, 8, 11 [β-Subunit of β-Conglycinin] 31, 32 [α-Subunit of β-Conglycinin] 33 [Glycinin A3B4 subunit] 34 [Glyg5_SoybnGlycinin] 36–39 [Mutant glycinin Subunit A1aB1b] 21 [Sucrose binding protein homolog S-64]	19 [Glyso Sucrose-binding protein] 21, 22 [Sucrose binding protein homolog S-64] 24 [β-Subunit of β-Conglycinin]	1–3, 26 [α-Subunit of β-Conglycinin] 7, 9, 10, 17, 18, 25, 27–30, 42 [β-Subunit of β-Conglycinin] 20, 23, 49 [Glyso Sucrose-binding protein] 40, 41 [Glyso Lectin] 45 [Glyso Glycinin] 46, 47 [Glycinin G4 subunit] 44 [Mutant glycinin A3B4] 43, 48 [Uncharacterized protein]

Note: * Higher = Spots protein presented in both conditions (cheesecloth and centrifuge separation) but the higher level of (abundance or quantity) in one condition versus other depending on the quantity of each protein spot using PDQuest analysis software in Table 2.

**Table 4:** Proteins extractability in soybean milk as affected by split and whole-seed.

Type of Seeds to Make Milk	Separation Techniques	Present Only in Split-Seed Extractions	Higher Level of Abundance in Split-Seed Extraction *	Present Only in Whole-Seed Extractions	Higher Level of Abundance in Whole-Seed Extractions *
Split versus whole	Cheesecloth	14, 15, 16, 24 [β-Subunit of β-Conglycinin] 20, 23 [Glyso Sucrose-binding protein] 13, 35 [Uncharacterized protein]	1–3 [α-Subunit of β-Conglycinin] 4–10, 17, 18 [β-Subunit of β-Conglycinin] 46, 47 [Glycinin G4 subunit] 36–39 [Mutant glycinin Subunit A1aB1b]	25, 27–29 [β-Subunit of β-Conglycinin] 21, 26, 30-32 [α-Subunit of β-Conglycinin] 33 [Glycinin A3B4 subunit] 48 [Uncharacterized protein] 49 [Glyso Sucrose-binding protein]	11, 42 [β-Subunit of β-Conglycinin] 41, 40 [Glyso Lectin] 34 [Glyg5_SoybnGlycinin] 45 [Glyso Glycinin] 12, 44 [Mutant glycinin A3B4] 43 [Uncharacterized protein]
Split versus whole	Centrifuge	14, 15, 16 [β-Subunit of β-Conglycinin] 35 [Uncharacterized protein]	4–11, 17, 18, 24 [β-Subunit of β-Conglycinin] 1–3 [α-Subunit of β-Conglycinin] 23 [Glyso Sucrose-binding protein] 21, 22 [Sucrose binding protein homolog S-64] 44 [Mutant glycinin A3B4] 45 [Glyso Glycinin]	25, 27–29 [β-Subunit of β-Conglycinin] 26, 30–32 [α-Subunit of β-Conglycinin] 33 [Glycinin A3B4 subunit] 48 [Uncharacterized protein] 49 [Glyso Sucrose-binding protein]	42 [β-Subunit of β-Conglycinin] 20 [Glyso Sucrose-binding protein] 34 [Glyg5_SoybnGlycinin] 36–39 [Mutant glycinin Subunit A1aB1b] 41, 40 [Glyso Lectin] 43 [Uncharacterized protein] 46, 47 [Glycinin G4 subunit]

Note: * Higher = Spots protein presented in both conditions (split and whole-seed milk extraction), but the higher level of abundance or quantity in one condition versus the other depending on the quantity of each protein spot using PDQuest analysis software in Table 2.

**Table 5:** List of the total protein concentration of soybean cheese for each condition.

Type of Soybean Seeds to Make Cheese	Separation Method	Total Protein (g/100 g of Cheese) Mean ± SD (n = 3)
Split	cheesecloth	21.26 ± 0.11
Split	centrifuge	26.80 ± 1.00
Whole	cheesecloth	27.62 ± 0.02
Whole	centrifuge	30.63 ± 0.20

SD = standard deviation. Number of replicates (n = 3).

**Table 6:** Yield and sensory analysis of soybean cheeses produce by vinegar from cultivar Bunya of soybean seeds (*Glycine max*)/cow’s milk and storage at 4 °C for one week.

Milk	Separation Methods	Yield (%)	Appearance	Color	Flavor	Texture	Overall Acceptability
Split	Cheesecloth	17.00 ± 0.70	3.66 ± 0.67	3.41 ± 0.68	3.55 ± 0.68	3.55 ± 0.57	3.76 ± 0.57
Split	Centrifuge	14.25 ± 0.35	2.97 ± 0.61	2.97 ± 0.61	3.00 ± 0.69	3.20 ± 0.66	3.31 ± 0.68
Whole	Cheesecloth	16.25 ± 0.33	2.60 ± 0.49	2.37 ± 0.49	2.40 ± 0.49	2.37 ± 0.49	2.60 ± 0.62
Whole	Centrifuge	13.50 ± 0.70	2.43 ± 0.50	2.53 ± 0.50	2.45 ± 0.49	2.30 ± 0.46	2.77 ± 0.67
cow’s milk	NSM	17.50 ± 0.70	4.17 ± 0.46	3.80 ± 0.48	3.83 ± 0.53	3.87 ± 0.57	4.07 ± 0.69

NSM: no separation method.

**Table 7:** MS/MS identification of differentiating proteins of the cultivar Bunya of soybean milk seeds (*Glycine max*). Matching has been achieved using Mascot sequence matching software (Matrix Science) with the taxonomy set to Viridiplanate (Green Plants). The spots are significantly different (*p* < 0.05) at PDQuest Bio-Rad.

NO	Protein	NCBI Accession Number	Database Theoretical * MW/PI	Sequence Coverage %	MOWES Score	Peptides
1	α-Subunit of β-Conglycinin (*Glycine max*)	gi|111278867	69,845/5.43	36	803	MITLAIPVNK, FNLRSRDPIY, ELAFPGSAKD, PGRFESFFLS, SYNLQSGDAL, STQAQQSYLQ, TPEKNPQLRD, RVPAGTTYYV, LDVFLSVVDM, VNPDNDENLR, NEGALFLPHF, VISQIPSQVQ
2	α-Subunit of β-Conglycinin (*Glycine max*)	gi|39718	70,263/5.12	32	927	NENLRLITLA, SEDKPFNLRS, LLPHFNSKAI, IPVNKPGRFE, EEGQQQGEQR, SFFLSSTEAQ, LQESVIVEIS, SGDALRVPSG, PQLRDLDIFL, DEDEDEEQDE, TTYYVVNPDN, SIVDMNEGAL
3	α-Subunit of β-Conglycinin (*Glycine max*)	gi|74271743	69,845/5.43	34	967	LFKNQYGHVR, MITLAIPVNK, NSKAIVVLVI, HGGKGSEEEQ, PGRFESFFLS, SNKLGKLFEI, NEGEANIELV, STQAQQSYLQ, TPEKNPQLRD, GFSKNILEAS, LDVFLSVVDM, VNPDNDENLR, YDTKFEEINK, VISQIPSQVQ, NEGALFLPHF
4	β-Subunit of β-Conglycinin (*Glycine Max*)	gi|1174098436	50,411/5.88	39	999	NNFGKFFEIT, PEKNPQLRDL, DIFLSSVDIN, NFLAGEKDNV, TYYLVNPHDH, KTISSEDEPF, EGALLLPHFN, NLRSRNPIYS, LAFPGSAQDV
5	β-Subunit of β-Conglycinin (*Glycine Max*)	gi|341603993	49,987/6.14	54	949	GRAILTLVNN, KFFEITPEKN, PQLRDLDIFL, AQPQQKEEGS, DYRIVQFQSK, GDAQRIPAGT, SSVDINEGAL, NNPFYFRSSN, PNTILLPHHA, TYYLVNPHDH, SEDEPFNLRS, SFQTLFENQN, DADFLLFVLS, RNPIYSNNFG
6	β-Subunit of β-Conglycinin (*Glycine Max*)	gi|21465631	47,947/5.67	56	888	IVQFQSKPNT, QRIPAGTTYY, QGFSHNILET, LSSVDINEGA, FYFRSSNSFQ, ILLPHHADAD, LVNPHDHQNL, SFHSEFEEIN, SSEDEPFNLR, FLLFVLSGRA, ILTLVNNDDR, PQLENLRDYR, DSYNLHPGDA, SSTQAQQSYL, NPQLRDLDIF
7	β-Subunit of β-Conglycinin (*Glycine Max*)	gi|341603993	49,987/6.14	48	911	GRAILTLVNN, KFFEITPEKN, DDRDSYNLHP, PQLRDLDIFL, AQPQQKEEGS, GDAQRIPAGT, SSVDINEGAL, NNPFYFRSSN, PNTILLPHHA, TYYLVNPHDH, SFQTLFENQN, DADFLLFVLS
8,9	β-Subunit of β-Conglycinin (*Glycine Max*)	gi|341603993	49,987/6.14	57	1074	GRAILTLVNN, KFFEITPEKN, DDRDSYNLHP, PQLRDLDIFL, AQPQQKEEGS, DYRIVQFQSK, SSVDINEGAL, NNPFYFRSSN, PNTILLPHHA, TYYLVNPHDH, SEDEPFNLRS, SFQTLFENQN, DADFLLFVLS, VLFGEEEEQR, RNPIYSNNFG
10	β-Subunit of β-Conglycinin (*Glycine Max*)	NP_0012368722	50,411/5.88	56	1058	GRAILTLVNN, EEQRQQEGVI, PEKNPQLRDL, YFVDAQPQQK, NNFGKFFEIT, KRSPQLENLR, DDRDSYNLHP, DYRIVQFQSK, DIFLSSVDIN, PNTILLPHHA, TYYLVNPHDH, KTISSEDEPF, SFQTLFENQN, DADFLLFVLS, NLRSRNPIYS, LAFPGSAQDV
11	β-Subunit of β-Conglycinin (*Glycine Max*)	gi|1174098436	50,411/5.88	52	1532	GRAILTLVNN, EEQRQQEGVI, NNFGKFFEIT, DDRDSYNLHP, PEKNPQLRDL, YFVDAQPQQK, GDAQRIPAGT, DIFLSSVDIN, NFLAGEKDNV, NNPFYLRSSN, PNTILLPHHA, TYYLVNPHDH, KTISSEDEPF, EGALLLPHFN, SFQTLFENQN, NLRSRNPIYS
12	Mutant glycinin A3B4 (*Glycine Max*)	gi|223649560	60,002/5.65	14	568	PGVPYWTYNT, GDEPVVAISL, IVTVEGGLSV, LDTSNFNNQL, DQNPRVFYLA, GFSKHFLAQS, FNEGDVLVIP, FNTNEDTAEK
13	Uncharacterized protein (*Glycine max*)	gi|947119133	54,647/5.30	24	560	WMYNNEDTPV, DSGAIVTVKG, VAVSIIDTNS, QEEENEGSNI, LENQLDQMPR, LSGFAPEFLK, RFYLAGNQEQ, EAFGVNMQIV, LIAVPTGVAW, RNLQGENEEE
14	β-Subunit of β-Conglycinin (*Glycine Max*)	gi|341603993	49,987/6.14	56	1308	GRAILTLVNN, QQEGVIVELS, KFFEITPEKN, DDRDSYNLHP, PQLRDLDIFL, AQPQQKEEGS, SSVDINEGAL, NNPFYFRSSN, PNTILLPHHA, PNTILLPHHA, TYYLVNPHDH, TYYLVNPHDH, SEDEPFNLRS, SFQTLFENQN, DADFLLFVLS, RNPIYSNNFG
15	β-Subunit of β-Conglycinin (*Glycine Max*)	gi|1174098436	50,411/5.88	58	1498	GRAILTLVNN, EEQRQQEGVI, NNFGKFFEIT, DDRDSYNLHP, PEKNPQLRDL, GDAQRIPAGT, DIFLSSVDIN, NFLAGEKDNV, NNPFYLRSSN, PNTILLPHHA, TYYLVNPHDH, KTISSEDEPF, EGALLLPHFN, DADFLLFVLS, NLRSRNPIYS, LAFPGSAQDV
16	β-Subunit of β-Conglycinin (*Glycine Max*)	gi|1174098436	50,411/5.88	56	1294	GRAILTLVNN, EEQRQQEGVI, NNFGKFFEIT, KRSPQLENLR, DDRDSYNLHP, PEKNPQLRDL, Y FVDAQPQQK, GDAQRIPAGT, NFLAGEKDNV, PNTILLPHHA, TYYLVNPHDH, KTISSEDEPF, EGALLLPHFN, NLRSRNPIYS, LAFPGSAQDV
17	β-Subunit of β-Conglycinin (*Glycine Max*)	gi|1174098436	50,411/5.88	33	762	LFKNQYGHVR, MITLAIPVNK, FNLRSRDPIY, ELAFPGSAKD, PGRFESFFLS, SYNLQSGDAL, STQAQQSYLQ, TPEKNPQLRD, RVPAGTTYYV, LDVFLSVVDM, LDVFLSVVDM, RNFLAGSKDN, VNPDNDENLR, NEGALFLPHF, VISQIPSQVQ,
18	β-Subunit of β-Conglycinin (*Glycine Max*)	gi|1174098436	50,411/5.88	37	913	RQFPFPRPPH, NENLRLITLA, SEDKPFNLRS, LLPHFNSKAI, PSQVQELAFP, IPVNKPGRFE, EEGQQQGEQR, RDPIYSNKLG, SFFLSSTEAQ, LQESVIVEIS, ESEDSELRRH, SGDALRVPSG, PQLRDLDIFL, DEDEDEEQDE, TTYYVVNPDN, SIVDMNEGAL
19	Glyso sucrose-binding protein (*Glycine Soja*)	gi|1169100901	57,954/6.08	43	933	AILEARAHTF, LSAFSWNVLQ, WWPFGGESKP, PSYHRISSDL, FAGKDNIVSS, VSPRHFDSEV, KPGMVFVVPP, LAMLHIPVSV, VGPDDDEKSW, LLQGIENFRL, GPGGRDPESV, GHPFVTIASN
20	Glyso sucrose-binding protein (*Glycine Soja*)	gi|1169100901	57,954/6.08	46	986	AILEARAHTF, LSAFSWNVLQ, WWPFGGESKP, PSYHRISSDL, FAGKDNIVSS, VSPRHFDSEV, KPGMVFVVPP, LAMLHIPVSV, VGPDDDEKSW, LLQGIENFRL, GPGGRDPESV, GHPFVTIASN
21	Sucrose binding protein homolog S-64 (*Glycine Max*)	gi|6179947	57,954/6.08	39	934	AILEARAHTF, HIPAGTPLYI, LSAFSWNVLQ, PSYHRISSDL, FAGKDNIVSS, VSPRHFDSEV, IHYNSHATKI, LDNVAKELAF, NYPSEMVNGV, LAMLHIPVSV, LGLVSESETE, STPGKFEEFF, FDRKESFFFP, LLQGIENFRL, KITLEPGDMI, GPGGRDPESV
22	Sucrose binding protein homolog S-64 (*Glycine Max*)	gi|6179947	55,799/6.32	22	476	SPRHFDSEVV, QTPKGKLERL, SHATKIALVM, GKFEEFFGPG, LQGNENFRLA, ITLEPGDMIH, GRDPESVLSA, ILEARAHTFV, FSWNVLQAAL, NIVSSLDNVA, QRSMSTIHYN
23	Glyso Sucrose-binding protein (*Glycine Soja*)	gi|1169100901	57,954/6.08	44	1135	AILEARAHTF, HIPAGTPLYI, LSAFSWNVLQ, VSPRHFDSEV, LDNVAKELAF, NYPSEMVNGV, LGLVSESETE, VGPDDDEKSW, FDRKESFFFP, LLQGIENFRL, KITLEPGDMI, GPGGRDPESV, GPGGRDPESV, FELPREERGR
24	β-Subunit of β-Conglycinin (*Glycine Max*)	gi|1174098436	50,411/5.88	58	1441	GRAILTLVNN, EEQRQQEGVI, NNFGKFFEIT, DDRDSYNLHP, PEKNPQLRDL, DIFLSSVDIN, NFLAGEKDNV, NNPFYLRSSN, PNTILLPHHA, TYYLVNPHDH, KTISSEDEPF, EGALLLPHFN, SFQTLFENQN, DADFLLFVLS, NLRSRNPIYS, LAFPGSAQDV
25	β-Subunit of β-Conglycinin (*Glycine Max*)	gi|1174098436	50,445/5.88	42	952	NNFGKFFEIT, DDRDSYNLHP, PEKNPQLRDL, YFVDAQPQQK, GDAQRIPAGT, DIFLSSVDIN, NFLAGEKDNV, TYYLVNPHDH, KTISSEDEPF, EGALLLPHFN, NLRSRNPIYS, LAFPGSAQDV
26	α-Subunit of β-Conglycinin (*Glycine max*)	gi|111278867	69,845/5.43	15	349	FNLRSRDPIY, ELAFPGSAKD, PGRFESFFLS, IENLIKSQSE, QLQNLRDYRI, STQAQQSYLQ, RNFLAGSKDN, GFSKNILEAS, YDTKFEEINK, RKTISSEDKP, VISQIPSQVQ
27	β-Subunit of β-Conglycinin (*Glycine Max*)	gi|1174098436	50,411/5.88	37	858	GRAILTLVNN, PVNKPGRYDD, EEQRQQEGVI, NNFGKFFEIT, KRSPQLENLR, DDRDSYNLHP, YFVDAQPQQK, GDAQRIPAGT, DIFLSSVDIN, EEEPLEVQRY, NFLAGEKDNV, EGALLLPHFN
28	β-Subunit of β-Conglycinin (*Glycine Max*)	gi|341603993	49,987/6.14	48	1155	GRAILTLVNN, KFFEITPEKN, DDRDSYNLHP, PQLRDLDIFL, DYRIVQFQSK, SSVDINEGAL, NNPFYFRSSN, PNTILLPHHA, SEDEPFNLRS, DADFLLFVLS, VLFGEEEEQR, RNPIYSNNFG
29	β-Subunit of β-Conglycinin (*Glycine Max*)	gi|341603993	49,987/6.14	42	578	GRAILTLVNN, KFFEITPEKN, NIELVGIKEQ, DDRDSYNLHP, PQLRDLDIFL, GDAQRIPAGT, SSVDINEGAL, PNTILLPHHA, TYYLVNPHDH, LLPHFNSKAI, DADFLLFVLS, VILVINEGDA
30	α-Subunit of β-Conglycinin (*Glycine max*)	gi|111278867	69,845/5.43	31	861	LFKNQYGHVR, MITLAIPVNK, VLFGREEGQQ, PGRFESFFLS, QGEERLQESV, STQAQQSYLQ, TPEKNPQLRD, GFSKNILEAS, LDVFLSVVDM, VNPDNDENLR, YDTKFEEINK, NEGALFLPHF
31	α-Subunit of β-Conglycinin (*Glycine max*)	gi|111278867	69,845/5.43	29	839	MITLAIPVNK, VLFGREEGQQ, FNLRSRDPIY, ELAFPGSAKD, QGEERLQESV, QLQNLRDYRI, SYNLQSGDAL, RVPAGTTYYV, LDVFLSVVDM, QEEQPLEVRK, VNPDNDENLR, YDTKFEEINK, RKTISSEDKP, NEGALFLPHF, VISQIPSQVQ
32	α-Subunit of β-Conglycinin (*Glycine max*)	gi|15425633	72,431/5.32	25	695	DALRVPSGTT, YYVVNPDNNE, NLRLITLAIP, DKPFNLRSRD, VNKPGRFESF, GQQQGEQRLQ, FLSSTEAQQS, ESVIVEISKE, FEITPEKNPQ
33	Glycinin A3B4 subunit (*Glycine Max*)	gi|126144646	57,663/5.78	31	629	MQQQQQQKSH, LRSPDDERKQ, HEDDEDEDEE, GGRKQGQHQQ, IVTVEGGLSV, EDQPRPDHPP, QEEEGGSVLS, QRPSRPEQQE, LHLPSYSPYP, GFSKHFLAQS, QMIIVVQGKG, GNPDIEHPET
34	Glyg5_SoybnGlycinin (*Glycine max*)	gi|121280	57,921/5.60	26	445	MQQQQQQKSH, LRSPDDERKQ, EDEEEDQPRP, SHLPSYLPYP, SHGKHEDDED, GGRKQGQHRQ, IVTVEGGLSV, DHPPQRPSRP, LQDSHQKIRH, QMIIVVQGKG, GNPDIEHPET, FNTNEDTAEK
35	Uncharacterized protein (*Glycine Max*)	gi|947119133	54,647/5.30	24	560	WMYNNEDTPV, DSGAIVTVKG, VAVSIIDTNS, QEEENEGSNI, LENQLDQMPR, LSGFAPEFLK, RFYLAGNQEQ, EAFGVNMQIV, LIAVPTGVAW, RNLQGENEEE
36	Mutant glycinin Subunit A1aB1b (*Glycine Max*)	gi|254029113	43,495/5.51	24	359	GHQSQKGKHQ, DKGAIVTVKG, QEEENEGGSI, GQSSRPQDRH, LSGFTLEFLE, RFYLAGNQEQ, HAFSVDKQIA, EFLKYQQEQG, KNLQGENEGE
37	Mutant glycinin Subunit A1aB1b (*Glycine Max*)	gi|254029113	43,495/5.51	27	402	GHQSQKGKHQ, DKGAIVTVKG, QEEENEGGSI, GQSSRPQDRH, LSGFTLEFLE, RPSYTNGPQE, RFYLAGNQEQ, EFLKYQQEQG, KNLQGENEGE
38	Mutant glycinin Subunit A1aB1b (*Glycine Max*)	gi|254029113	43,495/5.51	24	430	GHQSQKGKHQ, DKGAIVTVKG, QEEENEGGSI, GQSSRPQDRH, LSGFTLEFLE, RFYLAGNQEQ, EFLKYQQEQG, KNLQGENEGE
39	Mutant glycinin Subunit A1aB1b (*Glycine Max*)	gi|254029113	43,495/5.51	39	591	WMYNNEDTPV, GHQSQKGKHQ, VAVSIIDTNS, QEEENEGGSI, GQSSRPQDRH, LENQLDQMPR, LSGFTLEFLE, RPSYTNGPQE, RFYLAGNQEQ, LIAVPTGVAW, EFLKYQQEQG, KNLQGENEGE
40	Glyso Lectin (*Glycine Soja*)	gi|1236589326	309,009/5.65	39	545	ILQGDAIVTS, DASTSLLVAS, SGKLQLNKVD, RNSWDPPNPH, LVYPSQRTSN, ENGTPKPSSL, IGINVNSIRS, ILSDVVDLKT, IKTTSWDLAN, NKVAKVLITY
41	Glyso Lectin (*Glycine Soja*)	gi|1236589326	309,009/5.65	37	546	ILQGDAIVTS, DASTSLLVAS, RNSWDPPNPH, LVYPSQRTSN, IGINVNSIRS, ILSDVVDLKT, GRALYSTPIH, IKTTSWDLAN, NKFVPKQPNM, NKVAKVLITY
42	β-Subunit of β-Conglycinin (*Glycine Max*)	gi|1149122548	26,223/4.75	14	162	TQPGGASSVM, QSAATRNEQA, NPDATATPGG, VAASVAAAAR
43	Uncharacterized protein (*Glycine Max*)	gi|356535993	68,164/5.94	20	406	IVILMVTEGE, AQDIENLIKN, GKFYEITPEK, ANIELVGLKE, QRESYFADAQ, NPQLRDFDIL, QQQGEETREV, LNTVDINEGG, LLLPHYNSKA, VKELAFPAGS
44	Mutant glycinin A3B4 (*Glycine Max*)	gi|734345445	59,013/5.79	36	438	RLRQNIGQNS, VAAKSQSDNF, SPDIYNPQAG, EYVSFKTNDR, FSFLVPPQES, SITTATSLDF, PSIGNLAGAN, PALWLLKLSA, RVFDGELQEG, SLLNALPEEV, QYGSLRKNAM, GVLIVPQNFA, IQHTFNLKSQ
45	Glyso Glycinin (*Glyso Soja*)	gi|734345446	55,783/5.95	28	520	PALSWLRLSA, RVFDGELQEG, SLLNALPEEV, EFGSLRKNAM, FVPHYNLNAN, VAARSQSDNF, SIIYALNGRA, EYVSFKTNDT, FKFLVPPQES, PMIGTLAGAN
46	Glycinin G4 Subunit (*Glycine Max*)	gi|255224	63,641/5.38	9	264	VFKTHHNAVT, TLNSLTLPAL, PSEVLAHSYN, NNNPFSFLVP, GLLWGASKLV, QATKDDLTVY
47	Glycinin G4 subunit (*Glycine Max*)	gi|255224	63,641/5.38	7	209	FYNPKAGRIS, PKESQRRVVA, TLNSLTLPAL, SYLKDVFRAI, PSEVLAHSYN, NNNPFSFLVP
48	Uncharacterized protein (*Glycine Max*)	gi|947119133	54,647/5.30	10	260	DSGAIVTVKG, QEEENEGSNI, LSGFAPEFLK, RFYLAGNQEQ, EAFGVNMQIV, RNLQGENEEE
49	Glyso sucrose-binding protein (*Glycine Soja*)	gi|1169100901	57,954/6.08	43	933	AILEARAHTF, LSAFSWNVLQ, WWPFGGESKP, PSYHRISSDL, FAGKDNIVSS, VSPRHFDSEV, KPGMVFVVPP, LAMLHIPVSV, VGPDDDEKSW, LLQGIENFRL, GPGGRDPESV, GHPFVTIASN
50	Glyso Sucrose-binding protein (*Glycine Soja*)	gi|1169100901	69,845/5.43	46	986	AILEARAHTF, HIPAGTPLYI, LSAFSWNVLQ, PSYHRISSDL, FAGKDNIVSS, VSPRHFDSEV, IHYNSHATKI, LDNVAKELAF, NYPSEMVNGV, LAMLHIPVSV, LGLVSESETE, STPGKFEEFF, FDRKESFFFP, LLQGIENFRL, KITLEPGDMI, GPGGRDPESV
51	GlysoSucrose-binding protein (*Glycine Soja*)	gi|1174098436	50,411/5.88	45	1237	GRAILTLVNN, EEQRQQEGVI, NNFGKFFEIT, DDRDSYNLHP, PEKNPQLRDL, YFVDAQPQQK, GDAQRIPAGT, DIFLSSVDIN, NFLAGEKDNV, TYYLVNPHDH, KTISSEDEPF, EGALLLPHFN, NLRSRNPIYS, SFQTLFENQN
52	Glyso sucrose-binding protein (*Glycine Soja*)	gi|1169100901	57,954/6.08	44	1135	AILEARAHTF, HIPAGTPLYI, LSAFSWNVLQ, VSPRHFDSEV, LDNVAKELAF, NYPSEMVNGV, LGLVSESETE, VGPDDDEKSW, FDRKESFFFP, LLQGIENFRL, KITLEPGDMI, GPGGRDPESV, GPGGRDPESV, FELPREERGR
53	β-Subunit of β-Conglycinin (*Glycine Max*)	gi|1174098436	50,411/5.88	58	1441	GRAILTLVNN, EEQRQQEGVI, NNFGKFFEIT, DDRDSYNLHP, PEKNPQLRDL, DIFLSSVDIN, NFLAGEKDNV, NNPFYLRSSN, PNTILLPHHA, TYYLVNPHDH, KTISSEDEPF, EGALLLPHFN, SFQTLFENQN, DADFLLFVLS, NLRSRNPIYS, LAFPGSAQDV
54	β-Subunit of β-Conglycinin (*Glycine Max*)	gi|111278867	69,845/5.43	36	803	MITLAIPVNK, FNLRSRDPIY, ELAFPGSAKD, PGRFESFFLS, SYNLQSGDAL, STQAQQSYLQ, TPEKNPQLRD, RVPAGTTYYV, LDVFLSVVDM, VNPDNDENLR, NEGALFLPHF, VISQIPSQVQ
55	β-Subunit of β-Conglycinin (*Glycine Max*)	gi|1174098436	50,411/5.88	56	1294	GRAILTLVNN, EEQRQQEGVI, NNFGKFFEIT, KRSPQLENLR, DDRDSYNLHP, PEKNPQLRDL, YFVDAQPQQK, GDAQRIPAGT, NFLAGEKDNV, PNTILLPHHA, TYYLVNPHDH, KTISSEDEPF, EGALLLPHFN, NLRSRNPIYS, LAFPGSAQDV
56	β-Subunit of β-Conglycinin (*Glycine Max*)	gi|1174098436	50,411/5.88	58	1498	GRAILTLVNN, EEQRQQEGVI, NNFGKFFEIT, DDRDSYNLHP, PEKNPQLRDL, GDAQRIPAGT, DIFLSSVDIN, NFLAGEKDNV, NNPFYLRSSN, PNTILLPHHA, TYYLVNPHDH, KTISSEDEPF, EGALLLPHFN, DADFLLFVLS, NLRSRNPIYS, LAFPGSAQDV
57	β-Subunit of β-Conglycinin (*Glycine Max*)	gi|341603993	49,987/6.14	38	1308	GRAILTLVNN, QQEGVIVELS, KFFEITPEKN, DDRDSYNLHP, PQLRDLDIFL, AQPQQKEEGS, SSVDINEGAL, NNPFYFRSSN, PNTILLPHHA, TYYLVNPHDH, TYYLVNPHDH, SEDEPFNLRS, SFQTLFENQN, DADFLLFVLS, RNPIYSNNFG
58	β-Subunit of β-Conglycinin (*Glycine max*)	gi|1174098436	50,411/5.88	60	1554	GRAILTLVNN, EEQRQQEGVI, NNFGKFFEIT, DDRDSYNLHP, PEKNPQLRDL, YFVDAQPQQK, GDAQRIPAGT, DIFLSSVDIN, NFLAGEKDNV, NNPFYLRSSN, PNTILLPHHA, TYYLVNPHDH, KTISSEDEPF, EGALLLPHFN, SFQTLFENQN, NLRSRNPIYS
59	β-Subunit of β-Conglycinin (*Glycine max*)	gi|1174098436	50,411/5.88	45	1237	GRAILTLVNN, EEQRQQEGVI, NNFGKFFEIT, DDRDSYNLHP, PEKNPQLRDL, YFVDAQPQQK, GDAQRIPAGT, DIFLSSVDIN, NFLAGEKDNV, TYYLVNPHDH, KTISSEDEPF, EGALLLPHFN, NLRSRNPIYS, SFQTLFENQN
60	Mutant glycinin A3B4 (*Glycine Max*)	gi|223649560	60,002/5.65	14	368	PGVPYWTYNT, GDEPVVAISL, IVTVEGGLSV, LDTSNFNNQL, DQNPRVFYLA, GFSKHFLAQS, FNEGDVLVIP, FNTNEDTAEK
61	Uncharacterized protein (*Glycine max*)	gi|947119133	54,647/5.30	24	560	WMYNNEDTPV, DSGAIVTVKG, VAVSIIDTNS, QEEENEGSNI, LENQLDQMPR, LSGFAPEFLK, RFYLAGNQEQ, EAFGVNMQIV, LIAVPTGVAW, RNLQGENEEE
62	Glyso Glycinin (*Glycine Soja*)	gi|734345446	55,783/5.95	20	478	GGSQSQKGKH, EDKGAIVTVK, QQEEENEGGS, GQSSRPQDRH, ILSGFTLEFL, RPSYTNGPQE, RFYLAGNQEQ, EHAFSVDKQI, PDNRIESEGG, IYIQQGKGIF, AKNLQGENEG
63	Uncharacterized protein (*Glycine max*)	gi|947119133	54,647/5.30	18	501	DSGAIVTVKG, QEEENEGSNI, GQSSRPQDRH, LSGFAPEFLK, CQIQKLNALK, QKIYNFREGD, RFYLAGNQEQ, EAFGVNMQIV, RNLQGENEEE, PDNRIESEGG
64	Uncharacterized protein (*Glycine max*)	gi|947119133	54,647/5.30	16	459	DSGAIVTVKG, QEEENEGSNI, LSGFAPEFLK, EAFGVNMQIV, RFYLAGNQEQ, EAFGVNMQIV, RNLQGENEEE, EFLKYQQQQQ, PDNRIESEGG
65	Uncharacterized protein (*Glycine max*)	gi|351726399	27,863/6.92	35	361	FIGGTGYIGK, YPSEFGNDVD, FIVEASAKAG, RTHAVEPAKS, HPTFLLVRES, AFATKAKVRR, LGDGNPKAVF, ERIYVPEEQL
66	Glyso Glycinin (*Glycine Soja*)	gi|734345446	55,783/5.95	20	478	GGSQSQKGKH, EDKGAIVTVK, QQEEENEGGS, GQSSRPQDRH, ILSGFTLEFL, RPSYTNGPQE, RFYLAGNQEQ, EHAFSVDKQI, PDNRIESEGG, IYIQQGKGIF, AKNLQGENEG
67	Glyso Glycinin (*Glycine Soja*)	gi|734345446	55,783/5.95	17	410	PALSWLRLSA, RVFDGELQEG, SLLNALPEEV, RVLIVPQNFV, IQHTFNLKSQ, VAARSQSDNF, EYVSFKTNDT, FKFLVPPQES
68	Glyso Glycinin (*Glycine Soja*)	gi|734345446	55,783/5.95	25	517	PALSWLRLSA, RVFDGELQEG, SLLNALPEEV, EFGSLRKNAM, FVPHYNLNAN, VAARSQSDNF, SIIYALNGRA, EYVSFKTNDT, FKFLVPPQES, PMIGTLAGAN
69	Glyso Glycinin (*Glycine Soja*)	gi|734345445	59,013/5.79	24	446	RLRQNIGQNS, VAAKSQSDNF, SPDIYNPQAG, EYVSFKTNDR, FSFLVPPQES, SITTATSLDF, PSIGNLAGAN, PALWLLKLSA, RVFDGELQEG, SLLNALPEEV, QYGSLRKNAM, GVLIVPQNFA, IQHTFNLKSQ
70	α-Subunit of β-Conglycinin (*Glycine max*)	gi|111278867	69,845/5.43	32	762	LFKNQYGHVR, MITLAIPVNK, FNLRSRDPIY, ELAFPGSAKD, PGRFESFFLS, SYNLQSGDAL, STQAQQSYLQ, TPEKNPQLRD, RVPAGTTYYV, LDVFLSVVDM, LDVFLSVVDM, RNFLAGSKDN, VNPDNDENLR, NEGALFLPHF, VISQIPSQVQ
71	α-Subunit of β-Conglycinin (*Glycine max*)	gi|74271743	70,263/5.12	38	913	RQFPFPRPPH, NENLRLITLA, SEDKPFNLRS, LLPHFNSKAI, PSQVQELAFP, IPVNKPGRFE, EEGQQQGEQR, RDPIYSNKLG, SFFLSSTEAQ, LQESVIVEIS, ESEDSELRRH, SGDALRVPSG, PQLRDLDIFL, DEDEDEEQDE, TTYYVVNPDN, SIVDMNEGAL
72	α-Subunit of β-Conglycinin (*Glycine max*)	gi|111278867	69,845/5.43	36	803	MITLAIPVNK, FNLRSRDPIY, ELAFPGSAKD, PGRFESFFLS, SYNLQSGDAL, STQAQQSYLQ, TPEKNPQLRD, RVPAGTTYYV, LDVFLSVVDM, VNPDNDENLR, NEGALFLPHF, VISQIPSQVQ
73	Glyso Sucrose-binding protein (*Glycine Soja*)	gi|1169100901	57,954/6.08	33	761	AILEARAHTF, LSAFSWNVLQ, FAGKDNIVSS, VSPRHFDSEV, AALQTPKGKL, VFFNIKGRAV, LAMLHIPVSV, LGLVSESETE, STPGKFEEFF, VGPDDDEKSW, LLQGIENFRL, GPGGRDPESV, FELPREERGR
74	Glyso Sucrose-binding protein (*Glycine Soja*)	gi|1169100901	57,954/6.08	44	577	QHEEQDENPY, AILEARAHTF, HIPAGTPLYI, LSAFSWNVLQ, FAGKDNIVSS, IFEEDKDFET, IHYNSHATKI, KPGMVFVVPP, GHPFVTIASN, LGLVSESETE, VGPDDDEKSW, LLQGIENFRL, KITLEPGDMI, GPGGRDPESV, FELPREERGR
75	Glyso Sucrose-binding protein (*Glycine Soja*)	gi|1169100901	57,954/6.08	31	817	QHEEQDENPY, AILEARAHTF, LSAFSWNVLQ, FAGKDNIVSS, IFEEDKDFET, AALQTPKGKL, IHYNSHATKI, LDNVAKELAF, LGLVSESETE, LLQGIENFRL, FELPREERGR, SIFAISREQV
76	Glyso Sucrose-binding protein (*Glycine Soja*)	gi|1169100901	57,954/6.08	24	585	AILEARAHTF, LSAFSWNVLQ, FAGKDNIVSS, LDNVAKELAF, NYPSEMVNGV, LGLVSESETE, LLQGIENFRL, GPGGRDPESV
77	β-Subunit of β-Conglycinin (*Glycine Max*)	gi|1174098436	50,411/5.88	50	1093	EEQRQQEGVI, NNFGKFFEIT, EGDANIELVG, KRSPQLENLR, DDRDSYNLHP, PEKNPQLRDL, GDAQRIPAGT, DIFLSSVDIN, NFLAGEKDNV, TYYLVNPHDH, KTISSEDEPF, EGALLLPHFN, SKAIVILVIN, LAFPGSAQDV
78	β-Subunit of β-Conglycinin (*Glycine Max*)	gi|1174098436	50,411/5.88	55	1100	GRAILTLVNN, EEQRQQEGVI, NNFGKFFEIT, KRSPQLENLR, DDRDSYNLHP, PEKNPQLRDL, DIFLSSVDIN, NFLAGEKDNV, TYYLVNPHDH, KTISSEDEPF, EGALLLPHFN, SFQTLFENQN, SKAIVILVIN, LAFPGSAQDV
79	β-Subunit of β-Conglycinin (*Glycine Max*)	gi|1174098436	50,411/5.88	46	797	GRAILTLVNN, PVNKPGRYDD, EEQRQQEGVI, NNFGKFFEIT, GDAQRIPAGT, DIFLSSVDIN, NFLAGEKDNV, TYYLVNPHDH, KTISSEDEPF, EGALLLPHFN, NLRSRNPIYS, LAFPGSAQDV
80	β-Subunit of β-Conglycinin (*Glycine Max*)	gi|1174098436	50,411/5.88	45	880	GRAILTLVNN, EEQRQQEGVI, DDRDSYNLHP, PEKNPQLRDL, DIFLSSVDIN, NFLAGEKDNV, PNTILLPHHA, TYYLVNPHDH, KTISSEDEPF, EGALLLPHFN, SFQTLFENQN, DADFLLFVLS
81	β-Subunit of β-Conglycinin (*Glycine Max*)	gi|341603993	49,987/6.14	56	912	GRAILTLVNN, QQEGVIVELS, NIELVGIKEQ, DDRDSYNLHP, PQLRDLDIFL, QQKQKQEEEP, GDAQRIPAGT, NNPFYFRSSN, PNTILLPHHA, TYYLVNPHDH, LLPHFNSKAI, SFQTLFENQN, DADFLLFVLS, VLFGEEEEQR, VILVINEGDA
82	β-Subunit of β-Conglycinin (*Glycine Max*)	gi|341603993	49,987/6.14	52	896	GRAILTLVNN, QQEGVIVELS, KFFEITPEKN, NIELVGIKEQ, DDRDSYNLHP, PQLRDLDIFL, QQKQKQEEEP, GDAQRIPAGT, NNPFYFRSSN, TYYLVNPHDH, LLPHFNSKAI, SFQTLFENQN, VLFGEEEEQR, VILVINEGDA, GSAQDVERLL
83	GlysoGlycininA3B4subunit (*Glycine Soja*)	gi|126144646	57,663/5.78	25	566	LRSPDDERKQ, HEDDEDEDEE, IVTVEGGLSV, EDQPRPDHPP, QEEEGGSVLS, QRPSRPEQQE, LQDSHQKIRH, GFSKHFLAQS, GNPDIEHPET, FNTNEDTAEK
84	GlysoGlycininA3B4subunit (*Glycine Soja*)	gi|126144646	57,663/5.78	37	695	PGVPYWTYNT, LRSPDDERKQ, HEDDEDEDEE, GFSKHFLAQS, GDEPVVAISL, IVTVEGGLS, EDQPRPDHPP, LDTSNFNNQL, QEEEGGSVLS, QRPSRPEQQE, LHLPSYSPYP, DQNPRVFYLA, FNEGDVLVIP, GNPDIEHPET, FNTNEDTAEK
85	Uncharacterized protein (*Glycine max*)	gi|947119133	54,647/5.30	22	596	GGSQSQKGKQ, DSGAIVTVKG, QEEENEGSNI, GLRVTAPAMR, LSGFAPEFLK, RPSYTNGPQE, RFYLAGNQEQ, EAFGVNMQIV, EFLKYQQQQQ, RNLQGENEEE
86	Uncharacterized protein (*Glycine max*)	gi|947119133	54,647/5.30	18	475	DSGAIVTVKG, GGSQSQKGKQ, GLRVTAPAMR, RPSYTNGPQE, RFYLAGNQEQ, EAFGVNMQIV, RNLQGENEEE, EFLKYQQQQQ, IYIQQGKGIF, PDNRIESEGG
87	Uncharacterized protein (*Glycine max*)	gi|947119133	54,647/5.30	29	828	WMYNNEDTPV, DSGAIVTVKG, VAVSIIDTNS, QEEENEGSNI, LENQLDQMPR, LSGFAPEFLK, RPSYTNGPQE, RFYLAGNQEQ, EAFGVNMQIV, LIAVPTGVAW, RNLQGENEEE, PDNRIESEGG
88	Glyso Glycinin (*Glycine Soja*)	gi|734345446	55,783/5.95	19	539	EDKGAIVTVK, QQEEENEGGS, ILSGFTLEFL, RPSYTNGPQE, CQIQKLNALK, RPSYTNGPQE, RFYLAGNQEQ, EHAFSVDKQI, IYIQQGKGIF, EFLKYQQQQQ, AKNLQGENEG
89	Glyso Glycinin (*Glycine Soja*)	gi|734345446	55,783/5.95	18	473	EDKGAIVTVK, QQEEENEGGS, ILSGFTLEFL, RPSYTNGPQE, RFYLAGNQEQ, EHAFSVDKQI, PDNRIESEGG, IYIQQGKGIF, AKNLQGENEG
90	Glyso Glycinin (*Glycine Soja*)	gi|734345446	55,783/5.95	27	427	WMYNNEDTPV, EDKGAIVTVK, VAVSIIDTNS, QQEEENEGGS, LENQLDQMPR, ILSGFTLEFL, RPSYTNGPQE, RFYLAGNQEQ, LIAVPTGVAW, EFLKYQQQQQ, AKNLQGENEG
91	Glyso Elongation Factor (*Glycine Soja*)	gi|734402136	24,973/4.42	20	266	ASGLKKLDEY, IDALLRISGV, EESVRSVQME, LLPRSYITGY, GLLWGASKLV, QATKDDLTVY, PVGYGIKKLQ
92	Uncharacterized protein (*Glycine max*)	gi|947119133	54,647/5.30	13	274	DSGAIVTVKG, QEEENEGSNI, LSGFAPEFLK, RFYLAGNQEQ, EAFGVNMQIV, RNLQGENEEE
93	Glycinin G4 subunit (*Glycine Max*)	gi|255224	63,641/5.38	9	264	VFKTHHNAVT, TLNSLTLPAL, PSEVLAHSYN, NNNPFSFLVP
94	Glyso Glycinin (*Glycine Soja*)	gi|734345446	55,783/5.95	23	436	SLLNALPEEV, RVFDGELQEG, EFGSLRKNAM, RVLIVPQNFV, IQHTFNLKSQ, FVPHYNLNAN, VAARSQSDNF, SIIYALNGRA, EYVSFKTNDT, PMIGTLAGAN
95	Uncharacterized protein (*Glycine max*)	gi|356535993	68,164/5.94	30	409	IVILMVTEGE, AQDIENLIKN, GKFYEITPEK, ANIELVGLKE, QRESYFADAQ, NPQLRDFDIL, QQQGEETREV, LNTVDINEGG, LLLPHYNSKA, VKELAFPAGS, QEEENEGSNI
96	β-Subunit of β-Conglycinin (*Glycine max*)	gi|341603993	49,987/6.14	38	1035	MITLAIPVNK, VLFGREEGQQ, FNLRSRDPIY, ELAFPGSAKD, PGRFESFFLS, QGEERLQESV, NEGEANIELV, SYNLQSGDAL, STQAQQSYLQ, TPEKNPQLRD, GIKEQQQRQQ, LDVFLSVVDM
97	β-Subunit of β-Conglycinin (*Glycine max*)	gi|1174098436	50,411/5.88	39	1035	NENLRLITLA, EEINKVLFSR, SEDKPFNLRS, PSQVQELAFP, IPVNKPGRFE, EEGQQQGEQR, SFFLSSTEAQ, LQESVIVEIS, SGDALRVPSG, QSYLQGFSRN, PQLRDLDIFL, QQEQQQEEQP

* MW/PI = Molecular weight/Isoelectric point.
